# Percutaneous Cannulated Compression Screw Osteosynthesis in Phalanx Fractures: The Surgical Technique, the Indications, and the Results

**Published:** 2017-02-24

**Authors:** Eirini Liodaki, Tobias Kisch, Eike Wenzel, Peter Mailänder, Felix Stang

**Affiliations:** Department of Plastic Surgery, Hand Surgery and Burn Care Unit, University Hospital Schleswig-Holstein, Campus Lübeck, Lübeck, Germany

**Keywords:** cannulated screw, compression screw, percutaneous screw fixation, phalanx fracture, hand fracture

## Abstract

**Objective:** Fractures of metacarpals and phalanges are very common fractures, and there are a lot of treatment modalities. The purpose of the study was to describe the technique of percutaneous fixation of phalangeal fractures using a cannulated compression screw fixation system and its results. **Methods:** We conducted a prospective clinical study on 43 patients with different types of phalangeal fractures undergoing a percutaneous cannulated compression screw osteosynthesis. Parameters such as average operation time and clinical outcome were evaluated postoperatively. **Results:** Forty-three patients were treated using a percutaneous cannulated compression screw fixation system for phalanx fractures of the proximal (n = 26), middle phalanx (n = 16), or distal phalanx (n = 1). All fractures healed after 6 to 8 weeks except in 1 patient with secondary loss of reduction occurring 2.5 weeks after surgery. No infections were observed. The mean total active motion values were 247.56° ±16.16° and 244.35° ± 11.61° for the intra-articular fracture and 251.25° ± 19.86° for the shaft fractures; the mean Disabilities of the Arm, Shoulder, and Hand (DASH) score 3 months after the surgery was 1.67 ± 2.74. **Conclusions:** The advantages of this technique are the avoidance of an open procedure requiring extensive soft-tissue dissection with the risks of tendon adhesions and the achievement of interfragmentary compression. Because of the interfragmentary compression, it is superior to simple K-wires. With regard to indications, our primary focus was on unicondylar proximal interphalangeal joint fractures, shaft fractures, and simple oblique 2-fragment fractures.

Fractures of metacarpals and phalanges constitute between 14% and 28% of all visits to an emergency trauma department.^1^^,^^2^ Phalangeal fractures are nearly twice as common as metacarpal fractures. Most phalangeal fractures occur in the proximal phalanx, followed by the distal phalanx, and then the middle phalanx.^3^^-^5

Their treatment depends on the type of fracture, patient factors, and the surgeon's preference.^6^ Various treatment modalities used are percutaneous K-wire fixation, open reduction, and internal fixation with K-wires, screws, and microplates.^7^ Percutaneous fixation of phalangeal fractures is probably the most common technique in hand surgery. K-wires present a cost-effective option but typically require removal and do not provide compression. Another disadvantage of the K-wire fixation is the risk of pin track infection.^8^

Several types of phalangeal fractures such as unstable long spiral fractures, short oblique fractures, fractures with segmental bone loss or significant displacement, and intra-articular fractures benefit from internal fixation.^6^^,^^9^ Open reduction and internal fixation of phalangeal fractures with plates or lag screws (commonly used lag screw size in proximal phalanx fractures is 1.5 mm) are associated with tendon adhesions and finger stiffness.^8^ This kind of treatment provides a stable fixation, allowing an earlier and more intensive digital rehabilitation. However, postoperative finger stiffness is more likely to occur.^10^ Open reduction and internal fixation of hand fractures present a unique challenge due to their difficulty in managing small fragments without devascularizing them.^6^

While cannulated compressions screws have been known for a long time, they have not been used to treat phalangeal fractures because the available lengths and diameters were not suitable for this purpose.

For this study, we used 2 percutaneous cannulated compression screw fixation systems with a diameter of 1.7 mm (distributed as “Omnitech V17-system” by Biotech Ortho, a Wright Company, Salon de Provence, France; and “Small Headless Screws”- system by TriMed, Valencia, Calif).

The aim of this publication was to describe the surgical technique used, the indications, as well as our results and experience with these systems.

## MATERIALS AND METHODS

Between 2012 and 2015, we conducted a clinical study. Forty-three patients with phalangeal fractures undergoing percutaneous cannulated compression screw fixation were enrolled.

Inclusion criteria included acute fractures (within 2 weeks of injury) in skeletally mature patients. Patients with open fractures, patients with more than 1 fracture, skeletally immature patients, patients with fractures older than 2 weeks, and patients with fractures associated with osteoporosis were excluded.

All fractures treated were closed and considered unstable and therefore not amenable to conservative treatment. Unstable fractures have been defined as any rotational failure and angulation greater than 5° to 10° in the coronal plane and greater than 20° in the sagittal plane with pseudoclawing on clinical examination.

We treated all patients in the operating room under regional or general anesthesia with an upper-arm tourniquet. Surgical procedures were performed by 2 experienced hand surgeons of our department.

The following data were collected for each patient: age, sex, location of the fracture, type of the osteosynthesis with number and lengths of the screws used, operating time, duration of immobilization, complications, and time until recovery. Recovery is calculated by the regained total active motion (TAM) and the Disabilities of the Arm, Shoulder, and Hand (DASH) score. Excellent TAM is defined as 220° to 260° in the finger and 120° to 140° in the thumb.^11^ The follow-up protocol included radiological and clinical control on the first postoperative day, 2,4, and 6 weeks after the surgery, and clinical control after 3 and 6 months. If there was no fracture healing radiologically, radiological controls followed every second week till healing. The follow-up period is up to 6 months.

The radiological confirmation of fracture healing was made by the radiologist or by the surgeon and was featured by callus formation or invisible fracture line.

### Operative technique

**Figure 1:**
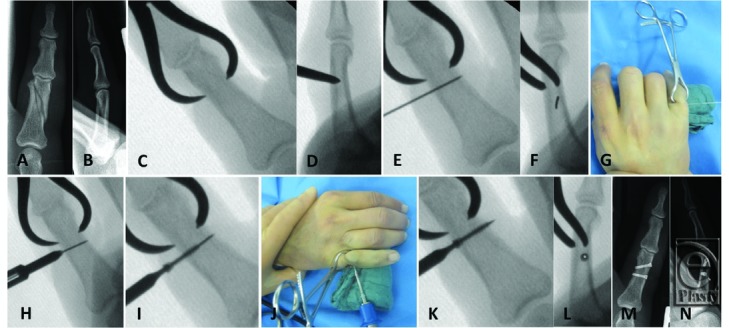
Operative technique. An oblique fracture of the proximal phalanx (a, b). After reposition and retention of the fracture with a reduction clamp (c, d), a 0.7-mm K-wire is inserted and secured into the opposite cortex (e-g). Defining the length and punching of the first corticalis is possible with the same device (h). The screw is then inserted over the K-wire (i-l). Postoperative control after inserting 2 screws in the same way (m, n).

**Figure 2:**
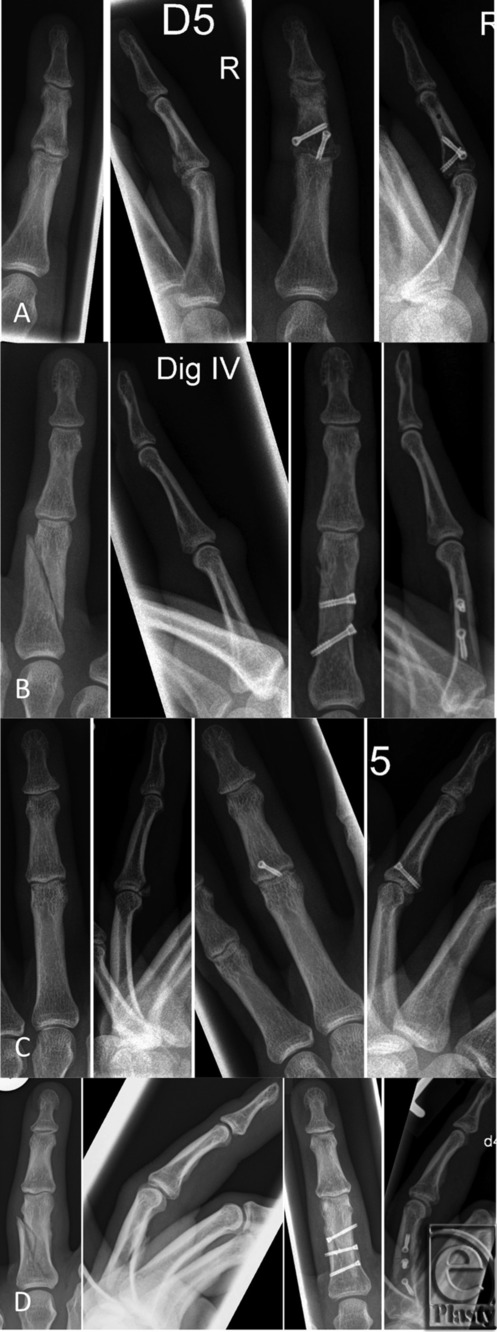
Four different cases (row a-d) with pre- and postoperative radiographs. (a) Intra-articular PIP joint fracture of the middle phalanx with 2 screws. (b) Typical extra-articular displaced oblique fracture treated with 2 screws. (c) Multiple fragments in the proximal phalanx treated with 3 screws, which is certainly a limitation for this type of osteosynthesis. (d) Mulifragment proximal phalanx fracture treated with 3 screws.

Surgery begins with the closed reduction of the fracture and correction of any rotational abnormality. After successful reduction of the fracture with the assistance of a percutaneous reduction clamp or temporary K-wiring, a 0.7-mm guiding K-wire is placed under fluoroscopic control; the tip of the K-wire has to be on the same level as the second cortex in order to determine the correct length. After proper placement, a mini skin incision of approximately 0.5 cm is placed and the correct length of the screw is determined by using the same device that has been used to punch the first cortex. To verify the accuracy of the measurement, fluoroscopic control of the right position of the length gauge, which has to have contact with the first cortex, is advised (see Video 1). The self-cutting, cannulated compression screw is then inserted via the K-wire. After the penetration of the second cortex, compression between bone fragments is applied, which should be seen in the fluoroscopic picture (Fig 1, see Video 2). After successful placement of the screw, K-wires and reduction clamps are removed. Where necessary, up to 3 screws were inserted (Fig 2*d*).

**Video 1:** After proper placement of the K-wire, a mini skin incision of approximately 0.5 cm is placed and the correct length of the screw is determined by using the same device that is used to punch the first cortex. To verify the accuracy of the measurement, fluoroscopic control of the right position of the length gauge, which has to have contact with the first cortex, is advised.

**Video 2:** The self-cutting, cannulated compression screw is inserted via the K-wire. After the penetration of the second cortex, compression between bone fragments is applied, which should be seen in the fluoroscopic picture.

Two finger splints, sparing the wrist, were initially applied for 2 weeks; however, with increasing experience, the immobilization was consecutively reduced down to 5 days in shaft fractures. Patients received physiotherapy with passive and careful active motion of all joints starting the first day after surgery.

## RESULTS

Between January 2012 and December 2015, a total of 43 patients (male: n = 32; female: n = 11) were treated with a percutaneous cannulated compression screw fixation system for phalanx fractures (Fig 2). The mean age of these patients was 37.98 ± 15.57 years. There were 23 intra-articular fractures (20 unicondylar, 3 bicondylar fractures), 20 extra-articular fractures, and fractures of the proximal (n = 26), middle (n = 16), or distal (n = 1) phalanx.

One to 3 cannulated compression screws were used in every fracture.

**Figure 3:**
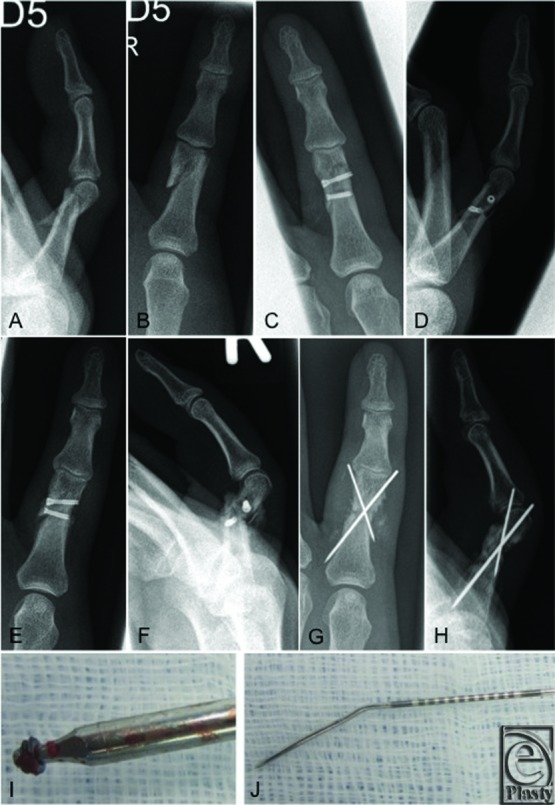
Presentation of a case. A 43-year-old patient undergoing a dislocated spiral fracture of the proximal phalanx of digiti minimi (a, b). The postoperative radiographs after the screw osteosynthesis (c, d). Note the missing head of 1 screw (c). Two weeks after the surgery, a secondary displacement of the fracture appeared (e, f) and was corrected by a new K-wire osteosynthesis (g, h). The broken screwhead (i) and the bent 0.7-mm K-wire used for the screw osteosynthesis (j).

Except for 1 patient with secondary loss of reduction 2.5 weeks after surgery (Fig 3), all fractures healed primarily after 6 to 8 weeks. No infections were observed. Implant removal was not required, with the exception of one patient who underwent a revision surgery due to the secondary loss of reduction. An excellent recovery with full range of motion was achieved after 6 weeks in 37 patients, a good recovery in 6 patients, and a fair recovery in 1 patient. The mean TAM values were 247.56° ± 16.16° and 244.35° ± 11.61° for the intra-articular fracture and 251.25° ± 19.86° for the shaft fractures; the mean DASH score 3 months after the surgery was 1.67 ± 2.74.

The mean duration of the operation was 32.25 ± 13.73 minutes.

## DISCUSSION

Treatment of phalangeal fractures depends on the type of fracture, degree of displacement, patient factors, and the surgeon's preference.

Despite the high number of patients with hand fractures, the literature does not provide guidance to the clinician on how specific phalangeal fractures should be treated.

Because of the broad spectrum of variation in fracture patterns and the many associated variables affecting the treatment and outcome of hand fractures, there are no randomized controlled trials.^3^

The main focus of this article is the percutaneous compression screw fixation of phalanx fractures. The 2 compression screw systems used are currently the thinnest screw available on the market and suitable for percutaneous fracture fixation of the phalanges even with small fragments. An advantage of these systems is the maintenance of reduction with the K-wire during the insertion of the cannulated screw. In addition, a stable osteosynthesis is achieved, allowing active movement of the affected joints. The surgical technique can be learned quickly and easily; nevertheless, the surgeon should select the patients very carefully.

Our main indications for this technique were simple, displaced oblique fractures of the proximal or middle phalanx and intra-articular fractures of the metacarpophalangeal joint or proximal interphalangeal joint (especially unicondylar fractures and shaft fractures with large, up to 3, fragments) (Fig 2).

In addition, treatment of the thumb is certainly possible; however, 2.0- or 2.3-mm cannulated screws of the same system are more adequate.

Critical points of the operation are to diagnose the correct fracture pattern, followed by exact closed reduction of the fragments that can be retained temporarily with reduction forceps before inserting the very thin and soft K-wires (Fig 3). Then, a correct measurement of the screw length is critical since removal and replacement of the screws may be difficult due to the very small screwhead with an increased risk of slippage of the screwdriver while turning it back.

Comparing this method with a conventional plate/screw osteosynthesis, the advantage of this technique is that an open procedure with the risks of scar formation and tendon adhesions can be avoided.^12^^,^^13^ With conventional compression screws based on the core-hole/gliding-hole principle, placement can only be attempted once, whereas the K-wires of the cannulated screws can be placed several times until the perfect position is confirmed by fluoroscopic control. Comparing the percutaneous cannulated compression screw fixation with traditional K-wires, both methods have almost the same degree of technical difficulty, but cannulated compression screws have the advantage of achieving interfragmentary compression without the need for later removal and operating time is only slightly increased. Common K-wires are much cheaper (about €0.50) than the compression screw (about €90), but time of immobilization for K-wire osteosynthesis is longer.^14^ This method can also be compared with the innovative compression wires of stainless steel, with 2 threads featuring different thread pitches according to the principle of the Herbert screw.^15^ These are indicated in simple transverse and short oblique shaft fractures of the proximal and middle phalanxes^15^ but not in intra-articular fractures. This operative technique offers compression to the fracture fragments as well, but the compression wires have to be removed after fracture healing and in this way a second surgery is needed.

We have already mentioned the complication of secondary loss of reduction in 1 patient. Our 43-year-old patient had a spiral fracture of the proximal phalanx of digiti minimi (Fig 3). During this operation, a screwhead broke while inserting it, most likely because of a material fault since we have only observed this problem once. A secondary loss of reduction of 40° occurred 2 weeks after surgery, retrospectively, as the result of a spiral fracture, which was not fixed by the screws in a proper manner. This case demonstrates the difficulty of placing the screws in the correct angle in complex fractures. After removal of the screws, an osteosynthesis with K-wires was performed.

The techniques used to treat phalangeal fracture (conservative, K-wires, compression cannulated screw, screw-pate osteosynthesis) are based on experiences gained in hand trauma practice. From our experience, we recommend the use of the cannulated compression screw fixation for phalangeal fractures, especially for intra-articular fractures, because of the aforementioned advantages of this technique, assuming a careful and patient-based indication.

In summary, we describe our experience with percutaneous cannulated compression screw fixation system for phalangeal fractures. The advantages of this technique are the avoidance of an open procedure leading to tendon adhesions, a short operating time, and interfragmentary compression with the possibility of earlier active movement.
